# Supplementary material for: Inhibiting the interaction between the mitochondrial receptor Tom70 and SARS CoV 2 Orf9b with small molecules
Source: bioRxiv. 2026 Apr 27:2026.04.27.721040. Preprint. [Version 1] doi: 10.64898/2026.04.27.721040 (PMC13142535; doi:10.64898/2026.04.27.721040)
Supplement: Supplement 2 [file NIHPP2026.04.27.721040v1-supplement-2.pdf]

bioRxiv preprint doi: https://doi.org/10.64898/2026.04.27.721040; this version posted April 27, 2026. The copyright holder for this preprint (which was not certified by peer review) is the author/funder, who has granted bioRxiv a license to display the preprint in perpetuity. It is made available under aCC-BY 4.0 International license.

Supplemental Figures

Supplemental Figure 1: Summary of DMSO and fragment soaked data sets

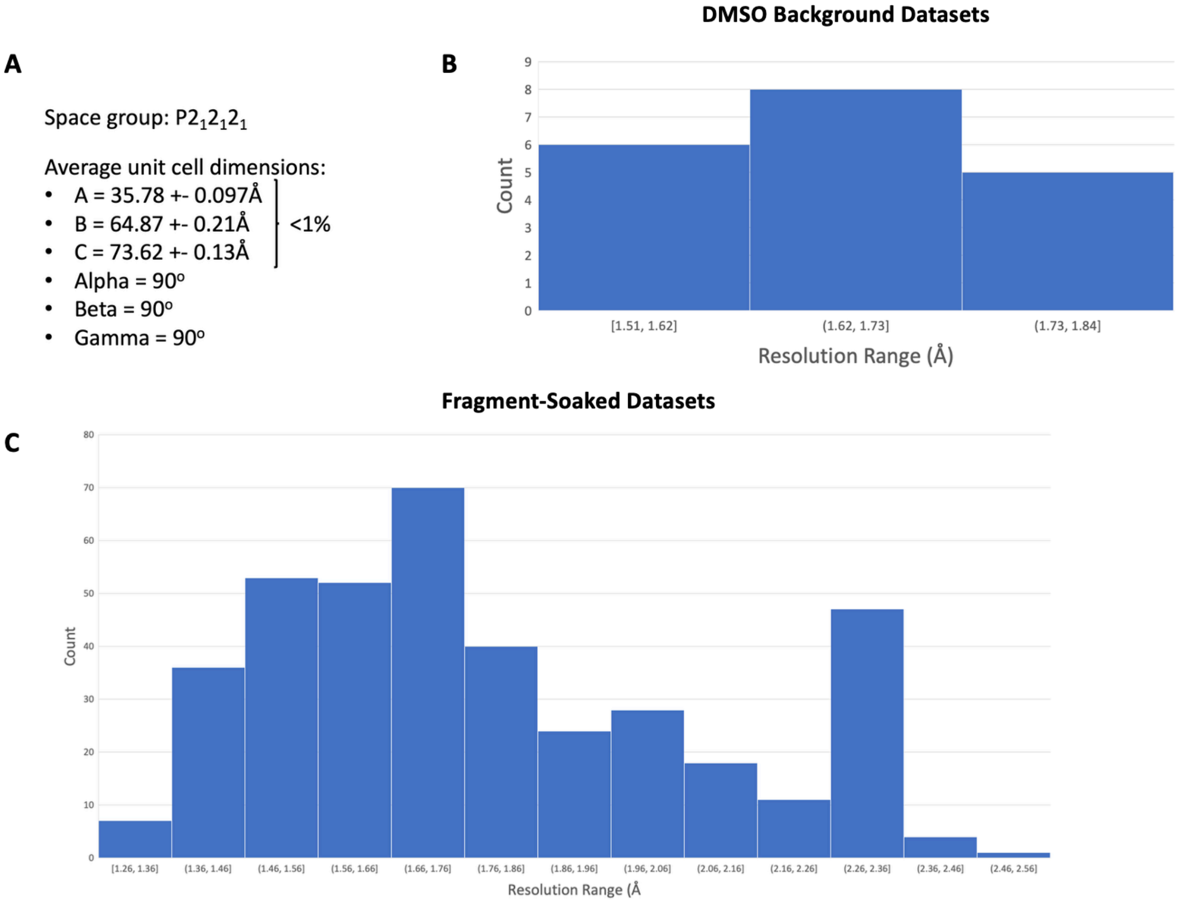

A. Orf9b homodimer crystal system unit cell parameters. All datasets collected showed less than 1% variation in unit cell dimensions.
B. Distribution of resolution ranges collected for DMSO soaked crystals used for PanDDA background maps.
C. Distribution of resolution ranges for fragment soaked datasets collected for conducting PanDDA analysis.

bioRxiv preprint doi: https://doi.org/10.64898/2026.04.27.721040; this version posted April 27, 2026. The copyright holder for this preprint (which was not certified by peer review) is the author/funder, who has granted bioRxiv a license to display the preprint in perpetuity. It is made available under aCC-BY 4.0 International license.

Supplemental Figure 2: Comparison of fragment binding poses in sites 1 and 2

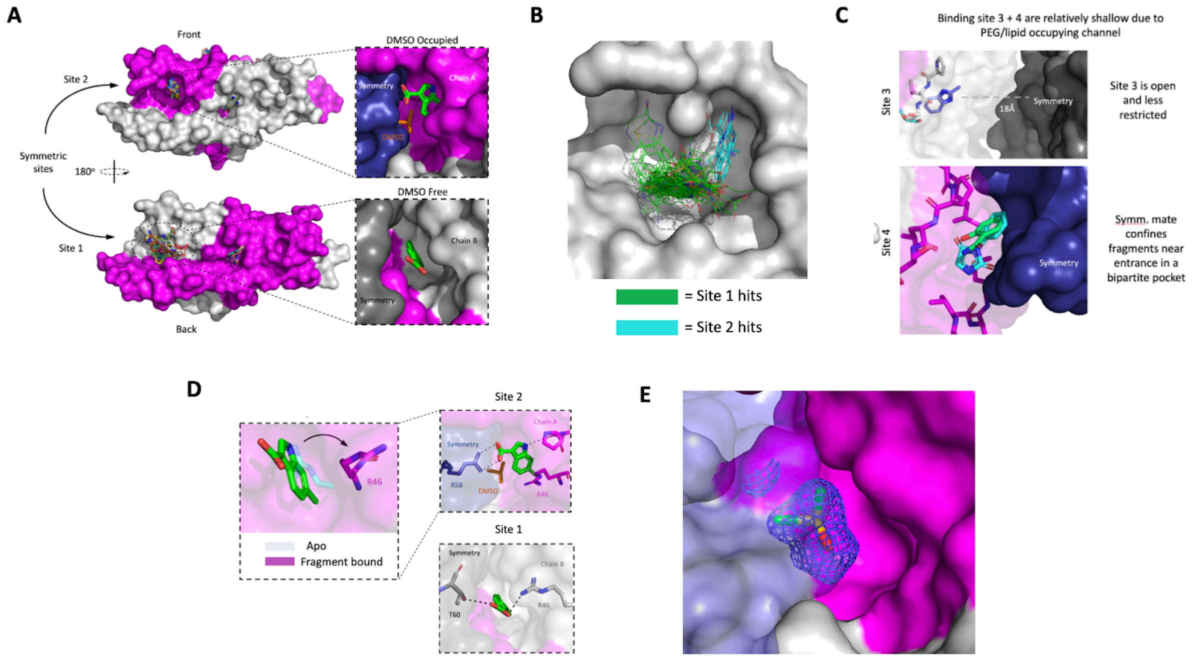

A. Comparison of fragment binding sites 1 and 2. Binding site 2 is partially occupied by a DMSO molecule and has fragments that are capable of forming favorable hydrogen bonds with the neighboring symmetry mate.
B. Superimposition of all modeled fragments from sites 1 onto site 2. Fragments at site 2 (cyan) bind in a region and orientation in the binding site that is distinct to site 1 fragments (green)
C. Comparison of neighboring symmetry mates at fragment binding sites 3 and 4. Binding site 3 fragments are approximately 22A away from the nearest symmetry mate whereas site 4 fragments are in close proximity to a symmetry mate near the central channel entrance.
D. Observed conformational change of R47 on chain B upon fragment binding. Differences in fragment binding poses observed in sites 1 and 2 with neighboring symmetry mate.
E. Electron density modeled as DMSO occupies part of binding site 2 reducing pocket size for fragment binding.

bioRxiv preprint doi: https://doi.org/10.64898/2026.04.27.721040; this version posted April 27, 2026. The copyright holder for this preprint (which was not certified by peer review) is the author/funder, who has granted bioRxiv a license to display the preprint in perpetuity. It is made available under aCC-BY 4.0 International license.

Supplemental Figure 3: Overview of FTMap predicted fragment hot-spots

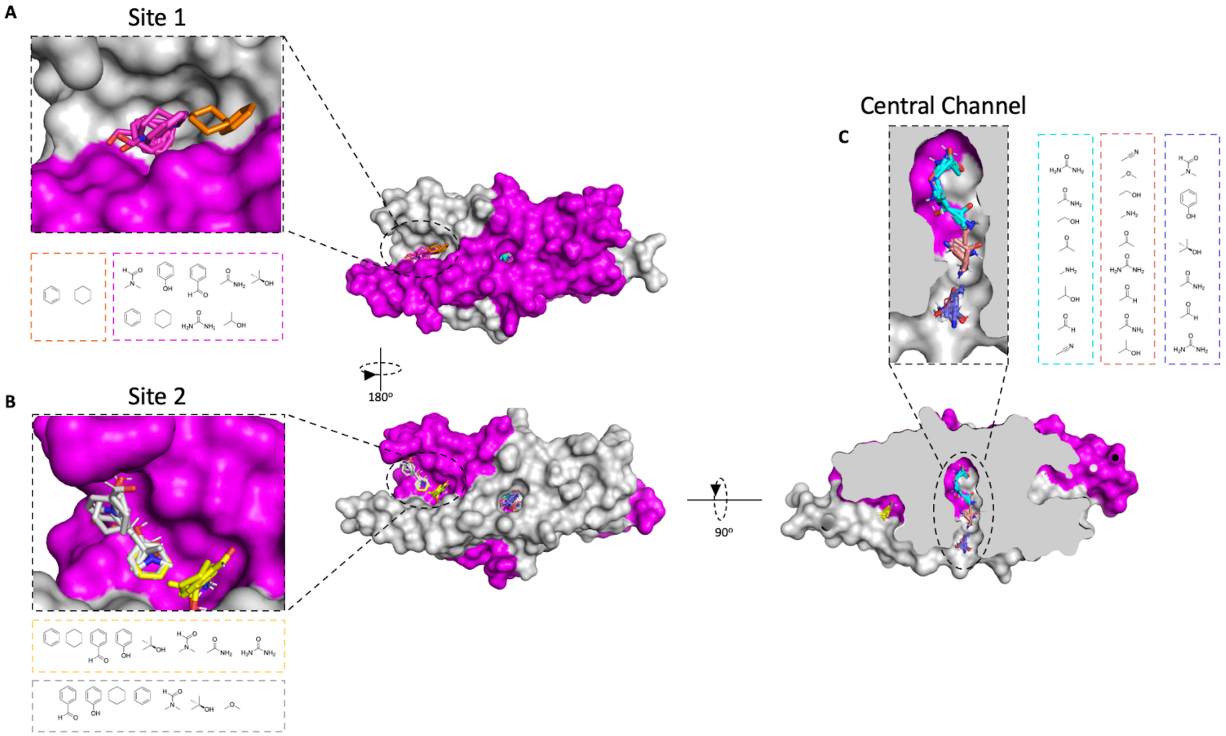

A. Distribution of FTMap probes bound at site 1 on the Orf9b homodimer.
B. Distribution of FTMap probes bound at site 2 on the Orf9b homodimer.
C. Distribution of FTMap probes bound to the central channel of the Orf9b homodimer.

bioRxiv preprint doi: https://doi.org/10.64898/2026.04.27.721040; this version posted April 27, 2026. The copyright holder for this preprint (which was not certified by peer review) is the author/funder, who has granted bioRxiv a license to display the preprint in perpetuity. It is made available under aCC-BY 4.0 International license.

Supplemental Figure 4: Lipidated analogs do not act on monomeric Orf9b or Tom70 directly

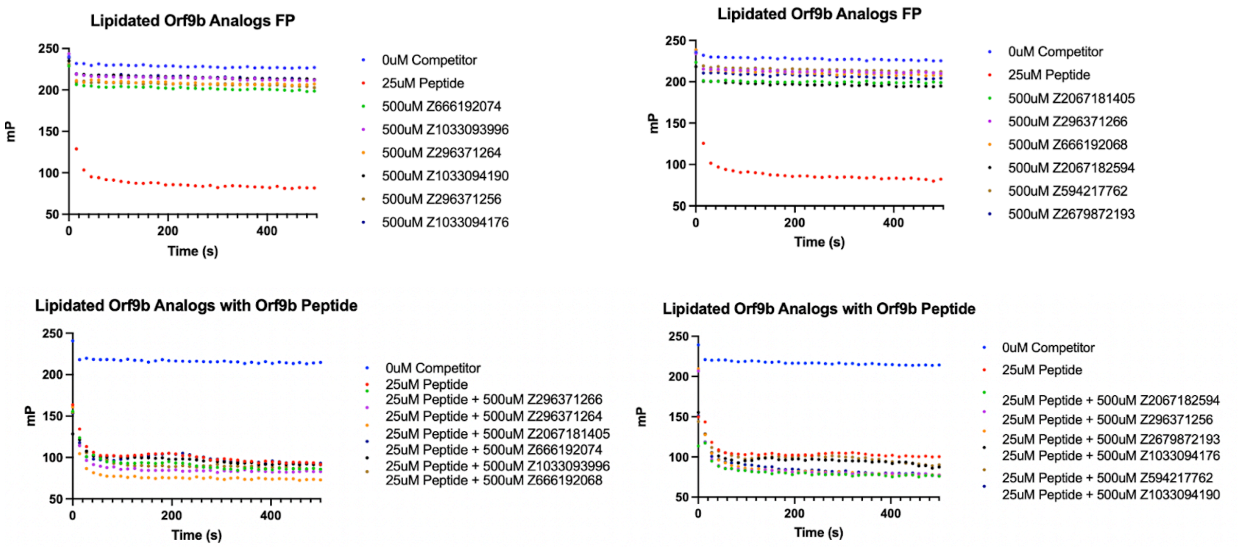

A. Lipidated Orf9b analogs do not compete with the fluorescent probe for binding to Tom70 in kinetic assay format. Control peptide exhibits a sharp decrease in FP signal over time as a positive control.
B. Lipidated Orf9b analogs do not interfere with Orf9b peptides from binding to Tom70 in kinetic assay format.

bioRxiv preprint doi: https://doi.org/10.64898/2026.04.27.721040; this version posted April 27, 2026. The copyright holder for this preprint (which was not certified by peer review) is the author/funder, who has granted bioRxiv a license to display the preprint in perpetuity. It is made available under aCC-BY 4.0 International license.

Supplemental Figure 5: Design of fluorescent Orf9b peptides and HTS screen.

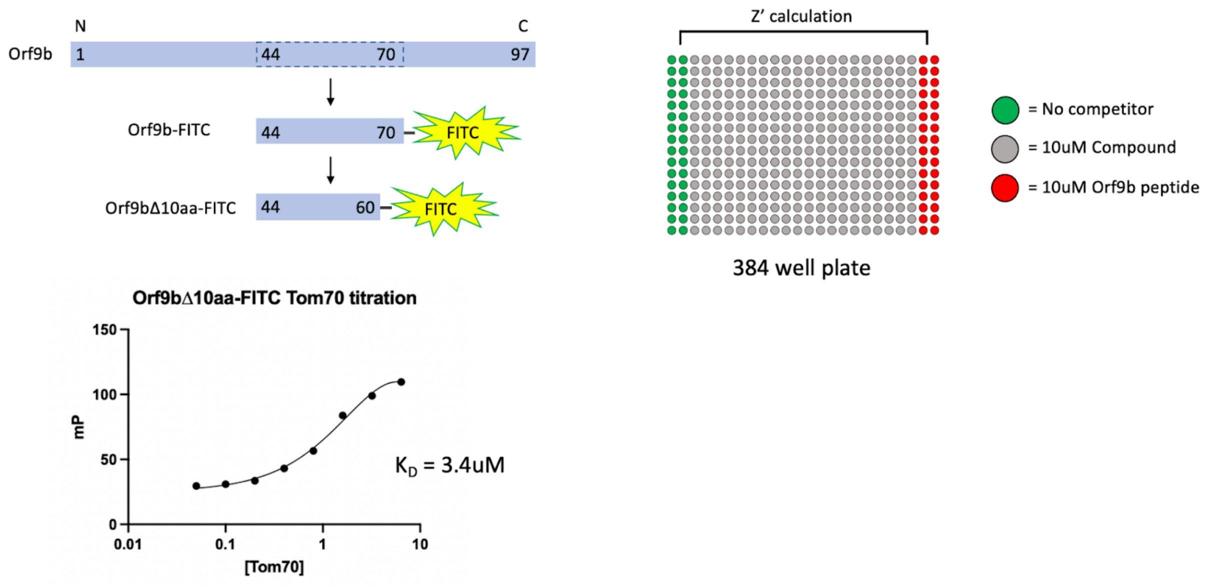

A. Design of fluorescent peptides derived from WT Orf9b. The structurally resolved residues of Orf9b bound to Tom70 (44-70) were used for generating C-terminally appended fluorescein peptides. A further 10 amino acids are truncated from the C-terminus of the Orf9b-FITC construct to generate the Orf9bΔ10aa-FITC fluorescent peptide used in the high throughput screen.
B. Titration of Tom70 against a fixed concentration of Orf9bΔ10aa-FITC was performed to identify the Kd. A non-linear regression single binding site model was used to determine the Kd of 3.4uM
C. Overview of a high-throughput screen set up. HTS was performed in 384 well plates with all wells containing the Tom70:Orf9bΔ10aa-FITC complex. Columns 1-2 contain only DMSO added and columns 23-24 contain 10uM of the Orf9b peptide as a positive control. Z' values were calculated from columns 1-2 and 23-24.

bioRxiv preprint doi: https://doi.org/10.64898/2026.04.27.721040; this version posted April 27, 2026. The copyright holder for this preprint (which was not certified by peer review) is the author/funder, who has granted bioRxiv a license to display the preprint in perpetuity. It is made available under aCC-BY 4.0 International license.

Supplemental Figure 6: Structure predictions for Tom70-small molecule binding sites

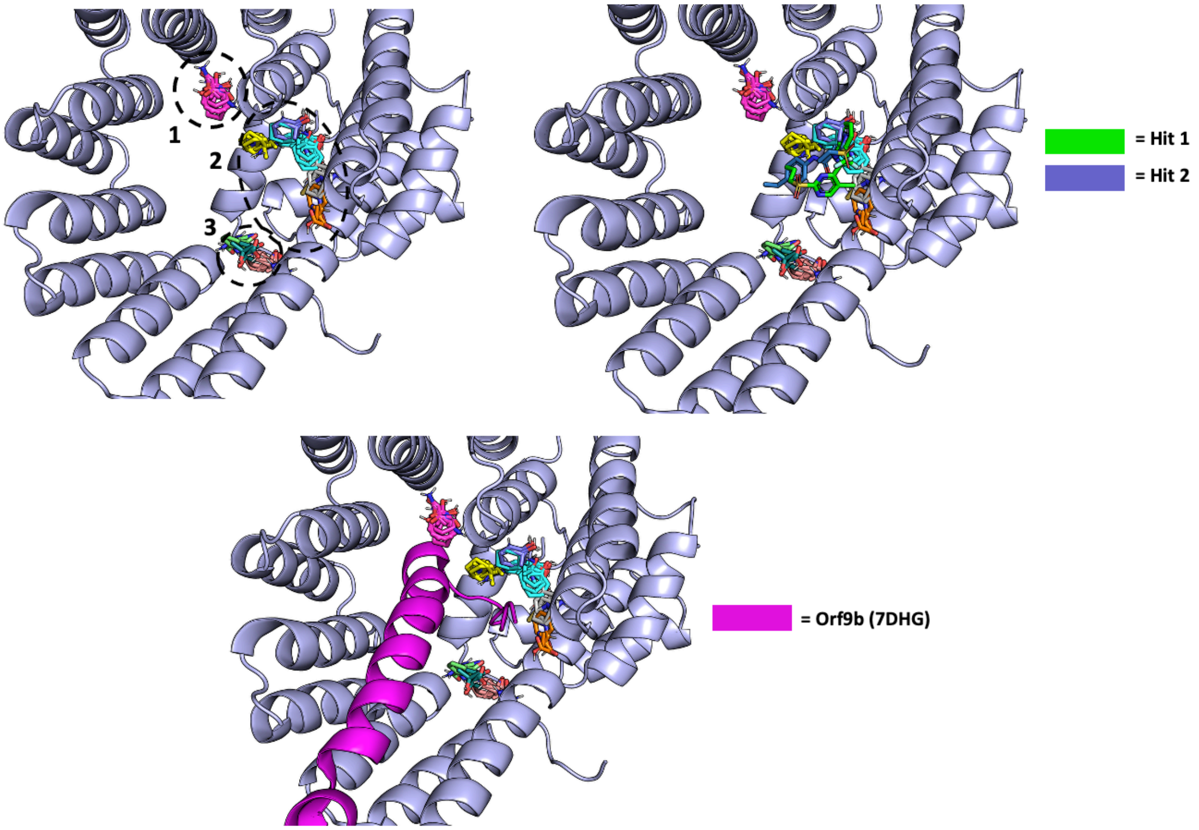

A. FTMap probe clusters within the Tom70 C-terminal binding domain. Clusters are grouped and number 1-3.
B. Superimposition of Chai-1 predicted binding locations for HTS Hits 1 and 2 with FTMap probe clusters.
C. Superimposition of Chai-1 predicted binding locations for HTS Hits 1 and 2 with FTMap probe clusters and Orf9b from the Cyro-EM structure of the Orf9b:Tom70 complex (PDB 7DHG).

bioRxiv preprint doi: https://doi.org/10.64898/2026.04.27.721040; this version posted April 27, 2026. The copyright holder for this preprint (which was not certified by peer review) is the author/funder, who has granted bioRxiv a license to display the preprint in perpetuity. It is made available under aCC-BY 4.0 International license.

Supplemental Figure 7: Dissociation Constants and Competition Binding Curves of Tom70 Analog Compounds

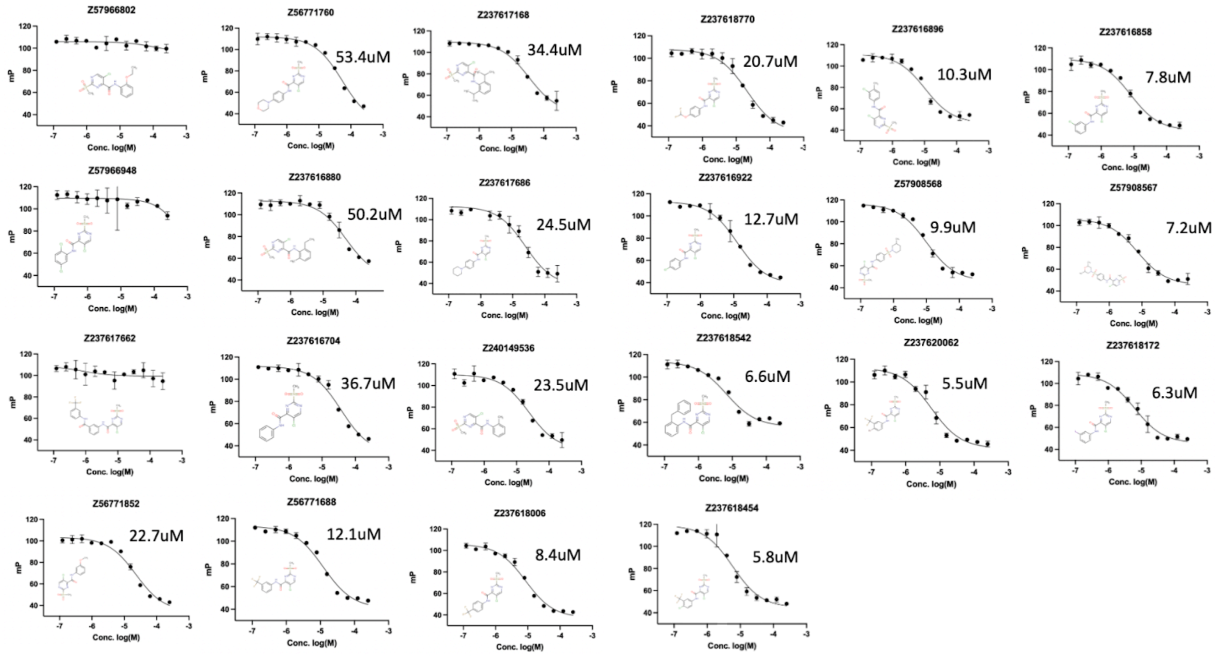

Competition binding curves of all tested Tom70 analog compounds in FP format. Error bars are for duplicate measurements with fitted curves shown using a one site fit for calculating Ki values. Structures of the compounds tested are shown in the bottom left.
